# Surficial N^+^ charge density indicating antibacterial capacity of quaternary ammonium resins in water environment

**DOI:** 10.1371/journal.pone.0239941

**Published:** 2020-09-30

**Authors:** Huaicheng Zhang, Aimin Li, Kaiqin Bian, Shanqi Shen, Peng Shi

**Affiliations:** State Key Laboratory of Pollution Control and Resource Reuse, School of the Environment, Nanjing University, Nanjing, China; Duke University Marine Laboratory, UNITED STATES

## Abstract

The antibacterial effects of quaternary ammonium resins (QARs) have been reported for decades, but there are few practical applications because of limited improvements in bactericidal capacity and the absence of an efficient antibacterial-indicating parameter. An in-situ determination method of surficial N^+^ groups for QARs, defined as surficial N^+^ charge density, was first established to merely quantify the exposed surficial quaternary ammonium groups (QAs). The mechanism of the new method depends on the tetraphenylboron sodium standard solution (TS), which is a colloidal solution with high steric hindrance, making it difficult to permeate into QARs and further react with the inner QAs. The results showed that the antibacterial efficacy of QARs correlates with the surficial N^+^ charge density with R^2^ > 0.95 (R^2^ of 0.97 for *Escherichia coli*, R^2^ of 0.96 for *Staphylococcus aureus*) but not with the strong-base group exchange capacity or zeta potential. Furthermore, the surficial N^+^ charge density was demonstrated efficient to indicate the antibacterial capacities against both gram-negative and gram-positive bacteria for commercial QARs, including acrylic, styrene and pyridine resin skeletons, especially for the QARs with similar skeletons and similar QAs. Based on the finding that the bactericidal groups merely involve the surficial QAs of QARs, this study proposes a new direction for improving the antibacterial capacity by enriching the surficial QAs and enhancing the bactericidal property of these surficial QAs, and provides a practicable synthesis with two-step quaternization.

## Introduction

It is indispensable to kill bacteria for the production of safe drinking water to protect against waterborne diseases. Common chemical disinfectants, such as chlorine, monochloramine and ozone [1, 2], unavoidably generate harmful disinfection byproducts (DBPs) in water disinfection, which closely correlate with cancer, miscarriage or birth defects [3, 4]. Meanwhile, ultraviolet (UV) radiation disinfection application is also limited because of bacterial regrowth via photoreactivation or dark repair [5–7]. More seriously, various tolerant bacteria have emerged to be resistant to these chemical disinfectants or antibiotics [8–10], posing threats to conventional water disinfection safety.

Quaternary ammonium resins (QARs) are not only timely separated to avoid secondary pollution and decrease eco-toxicological effects [11, 12] but are also efficient in removing resistant pathogens and antibiotic resistant genes (ARGs) in drinking water disinfection [13, 14], in comparison with soluble disinfectants. The bactericidal mechanism of QARs mainly includes: capturing bacteria by electrostatic attraction of N^+^ positive charges, permeating and destroying the cell membrane through hydrophobic alkyl chains [15], causing leakage of cytoplasm, and resulting in bacterial death with a changed cell shape [16, 17]. However, there has always been lack of an efficient and simple parameter for QARs to indicate their antibacterial capacity. Although quaternary ammonium groups (QAs) are demonstrated as biocidal groups and their quantitative methods [18–20], such as chemical titration, potentiometric titration, spectrophotometry, high-performance liquid chromatography (HPLC), gas chromatography (GC) or -mass spectrometry (MS), have been developed for various environmental samples, it is difficult to copy and analyze the QAs in insoluble and nonextractable solid materials. Charge density was used to quantify the surficial QAs grafted on glass slides and correlate with the bactericidal property, and an antibacterial charge-density threshold was also found to greatly influence antibacterial capacity [21, 22]. In fact, the surficial charge-density values were their total charge densities because of the good penetrability of fluorescein dye (for the quantification of charge density) [23]. Zeta potential was also adapted to clarify that the QARs with long-alkyl QAs exhibited higher antibacterial effects than those with short-alkyl QAs, inferring that the latter were incapable to overcome the repulsion of positively charged polymers [24]. However, Jie found an inconsistent result that the antibacterial polymeric resin with a higher zeta potential value exhibited a weaker bactericidal ability [25], which proves that it is unqualified as an antibacterial-indicating parameter. In addition, the zeta potential detection was always only applied in colloidal solutions, making it difficult to be detected in situ for large-particle insoluble materials (resins).

In this study, the surficial N^+^ charge density of QARs and its in-situ detection method was first proposed, which merely quantifies the surficial N^+^ groups of QARs. We detected the surficial N^+^ charge density, strong-base group exchange capacity (“exchange capacity” for short), charge density and zeta potential of QARs and analyzed their relevance to the antibacterial capacity, to establish an efficient antibacterial-indicating parameter. The surficial N^+^ charge density was demonstrated more suitable to indicate the antibacterial capacity against both gram-negative and gram-positive bacteria for QARs than the exchange capacity, better than the reported charge density or zeta potential. Moreover, the method applicability of the surficial N^+^ charge density was tested in commercial resins, including acrylic, styrene and pyridine resin skeletons. To our best knowledge, this is the first study to establish the new antibacterial-indicating parameter of the surficial N^+^ charge density, demonstrate its relevance to the antibacterial capacity and finish its application research in commercial QARs. As an in-situ antibacterial-indicating parameter, the surficial N^+^ charge density will provide significant guidance in synthesizing a highly efficient antibacterial resin or in selecting a best antibacterial resin from different QARs.

## Materials and methods

Poly(4-vinylpyridine) resin (25% cross-linkage, 10‒50 mesh) was purchased from Sigma-Aldrich Company in Shanghai, China. Commercial resins of D201, D205, D213, D314, D319 and D730 were provided by Zhengguang Industrial Co., Ltd. in Zhejiang, China. Tetraphenylboron sodium standard solution (TS) was purchased from Huaian Huake Chemical Co., Ltd. in Jiangsu, China. Iodomethane, 1-iodohexane, methanol, absolute ethanol, acetone, acetonitrile, cetyltrimethylammonium bromide (CTAB), formaldehyde solution (33 wt.%), sodium hydroxide and titan yellow were all chased from J&K Company in Beijing, China. All the above reagents were of analytical grade, and used without further purification. *Escherichia coli* (*E*. *coli*, ATCC8099) and *Staphylococcus aureus* (*S*. *aureus*, ATCC25923) were purchased from Guangdong Huankai Microbial Sci. & Tech. Co., Ltd. in Guangzhou, China.

### Preparation of Py-1C and Py-6C

The poly (4-vinylpyridine) resin with a pyridine ring grafting one methyl (Py-1C) was synthesized as follows [26]: 50 g of poly (4-vinylpyridine) resin (Py-0), 150 g of iodomethane and 150 mL of absolute ethanol were added into a 1000-mL, three-necked flask with a mechanical stirrer. The reaction was held at 38°C, under reflux for 48 h. Unreacted reagents were removed several times via extraction with methanol, acetone and absolute ethanol, respectively. Then, the new resin was activated by 15% (wt.%) sodium chloride solution and washed 5 times with ultrapure water. Similarly, the poly(4-vinylpyridine) resin with a pyridine ring grafting one hexyl (Py-6C) was prepared using 1-iodohexane.

The synthetic and characteristic information of the above resins of Py-1C and Py-6C are recorded in S1 Table in S1 File.

### Determination of surficial N+ charge density

Principles: The method theory is that the tetraphenylboron sodium standard solution (TS) reacts with soluble quaternary ammonium compounds (QACs) to produce white precipitates in alkaline media. The excessive TS can be titrated by cetyltrimethylammonium bromide (CTAB), and then the remaining CTAB combines with titan yellow to generate pink compounds at the titration endpoint. This theory was introduced and demonstrated by Li [27], to efficiently detect water-soluble QACs, in comparison with a gravimetric method.

Establishment of method: Comparing different method steps, the optimal detection of “surficial N^+^ charge density” for QARs was first established as method B1 (Table 1) as follows: 1 g of the QARs, 5 drops of sodium hydroxide (20 wt.%), 10 mL of TS (0.02 mol/L), 5 mL of formaldehyde solution, and 5 drops of titan yellow (0.1 wt.%) were added to a 250-mL flask containing 100 mL of ultrapure water. The flask was blended slowly for several seconds, and then the reaction was allowed to stand for 40 min. Finally, the CTAB (0.01 mol/L) was used to titrate the excessive TS to the endpoint, with *V*_*2*_ recorded. In addition, the water retention capacity of QARs was determined according to standard operating procedures [28].

**Table 1:** Comparison of different detection steps in surficial N^+^ charge density.

Methods	Step 1	Step 2	Step 3	Step 4	Step 5
**A1**	adding resin and TS	*standing* for 40 min	adding *sodium hydroxide*, formaldehyde solution and titan yellow	titrating with CTAB	
**A2**	adding resin and TS	*shaking* for 40 min	adding *sodium hydroxide*, formaldehyde solution and titan yellow	titrating with CTAB	
**B1**	adding resin, TS, *sodium hydroxide*, formaldehyde solution and titan yellow	*standing* for 40 min	titrating with CTAB		
**B2**	adding resin, TS, *sodium hydroxide*, formaldehyde solution and titan yellow	*shaking* for 40 min	titrating with CTAB		
**C**	adding resin and TS	*shaking* for 40 min	adding *sodium hydroxide*, formaldehyde solution and titan yellow	*shaking* for 40 min	titrating with CTAB

A different reaction condition is “step 2” in the methods of A1 and A2 (B1 and B2); an earlier alkaline reaction condition is built in method B than in method A (step 1); method C is a combination of method A2 and method B2. TS: tetraphenylboron sodium standard solution; CTAB: cetyltrimethylammonium bromide.

Calculation formula:

$$\sigma =\frac{\left(C_{1}\times V_{1}–C_{2}\times V_{2}\right)}{m\times (1–X)}$$

*σ* is the surficial N^+^ charge density (mmol/g). *C*_*1*_ and *C*_*2*_ are the concentrations of TS and CATB solutions respectively (mol/L); *V*_*1*_ and *V*_*2*_ are the volumes of TS and CATB solutions respectively (mL); m is the weight of the test sample (g). *X* is the water retention capacity of the test sample (%).

### Determination of strong-base group exchange capacity

Method 4.2 of “Determination of exchange capacity of strong basic anion exchange resins in chloride form” [29] is used to determine the strong-base group exchange capacities of QARs (“exchange capacities of QARs” for short). In short, QARs are added into a 10-mL exchange column, and then fully exchanged by Na_2_SO_4_ solution (0.5 mol/L) to release the chloride ions (Cl^‒^) combined with QARs. Finally, the silver nitrate solution (0.1 mol/L) is used to titrate the chloride ions to the endpoint, with *V*_*2*_ recorded. Similarly, the titration volume of the blank control group is recorded as *V*_*1*_.

Calculation formula:

$$Q=\frac{(V_{2}–V_{1})C_{1}}{m(1–w)}$$

*Q* is the strong-base group exchange capacity (mmol/g); *V*_*1*_ is the titration volume of the blank control group (mL); *V*_*2*_ is the titration volume of the test sample (mL); *C*_*1*_ is the concentration of silver nitrate solution (mol/L); *m* is the weight of the test sample (g); *w* is the water retention capacity of the test sample (%).

### Disinfection assays

The antibacterial properties of QARs were evaluated by *E*. *coli* (ATCC8099) and *S*. *aureus* (ATCC25923), which are representative of gram-negative and gram-positive bacteria, respectively. The two bacteria were cultured with the Luria-Bertani (LB) broth for growth and the nutrient agar for number counting. The bacterial concentration was controlled to an optical density (OD) of approximately 1.20 at 600 nm, corresponding to approximately 10^9^ colony-forming units per milliliter (CFU/mL). For disinfection experiments, the bacterial suspension was diluted to approximately 10^5^ CFU/mL in a 250-mL flask containing 100 mL sterile water and 1 mL QARs (i.e., 100 bed volume (BV)). To ensure antibacterial equilibrium, the flasks were shaken at 200 revolutions per minute (rpm) and 20°C for 1 h. Then, the viable bacteria were diluted to a proper concentration for plate counting on the nutrient agar medium. Finally, the antibacterial efficacy was estimated by the following calculation formula:

$$e=\frac{C_{0}–C_{1}}{C_{0}}\times 100\%.$$

*e* is the antibacterial efficacy (%). *C*_*0*_ is the viable bacteria in the blank control group (CFU/mL); *C*_*1*_ is the viable bacteria in the experimental group (CFU/mL). In this study, each group of experiments was conducted in triplicate to improve reliability.

### Statistical analysis

The antibacterial efficacy and surficial N^+^ charge density detection value, expressed as the mean ± standard error, were calculated using Microsoft Excel 2016 (Microsoft, USA). Correlations were analyzed using Pearson’s correlation coefficients (R) by Origin 8.5 (OriginLab, USA).

### Analysis and characterization

The FT-IR spectra of the samples were observed by a Fourier-transform infrared spectrometer (NEXUS870; Thermo Fisher Scientific, USA). Particle sizes were detected by a granulometer (Mastersizer3000; Malvern, England). The UV-vis spectrometer UV-1800 (Shimadzu, Japan) was used to record the optical density (OD) of bacterial liquids at the wavelength of 600 nm. The TOC analyzer multi N/C 3100 (Analytikjena, Germany) was used to measure the total organic carbon (TOC) and total nitrogen (TN) of water samples. The basis of the BET model of NOVA 3000e (Quantachrome, America) was used to determine the specific surface area and average pore size of resins. The selected samples were investigated by a scanning electron microscopy (SEM) (Quanta 250 FEG; FEI, USA).

## Results and discussion

### Synthesis and characteristics of QARs

The FT-IR analyses for Py-1C and Py-6C were conducted within a band range of 4000–500 cm^-1^. The FT-IR spectra (S1 Fig in S1 File) confirm the presence of QAs in QARs with the vibration peak of C‒N 1172 cm^-1^, approaching that of the 1040 cm^-1^ reported by Zhang [16]. Moreover, the exchange capacities of Py-1C and Py-6C could be detected evidently, which indicated that there were plentiful chloride ions resulting from the quaternization of QARs (one N^+^ group combines with one Cl^‒^). The above results demonstrate that QAs were grafted onto QARs successfully.

### Establishment of detection method of surficial N+ charge density

**Fig 1:**
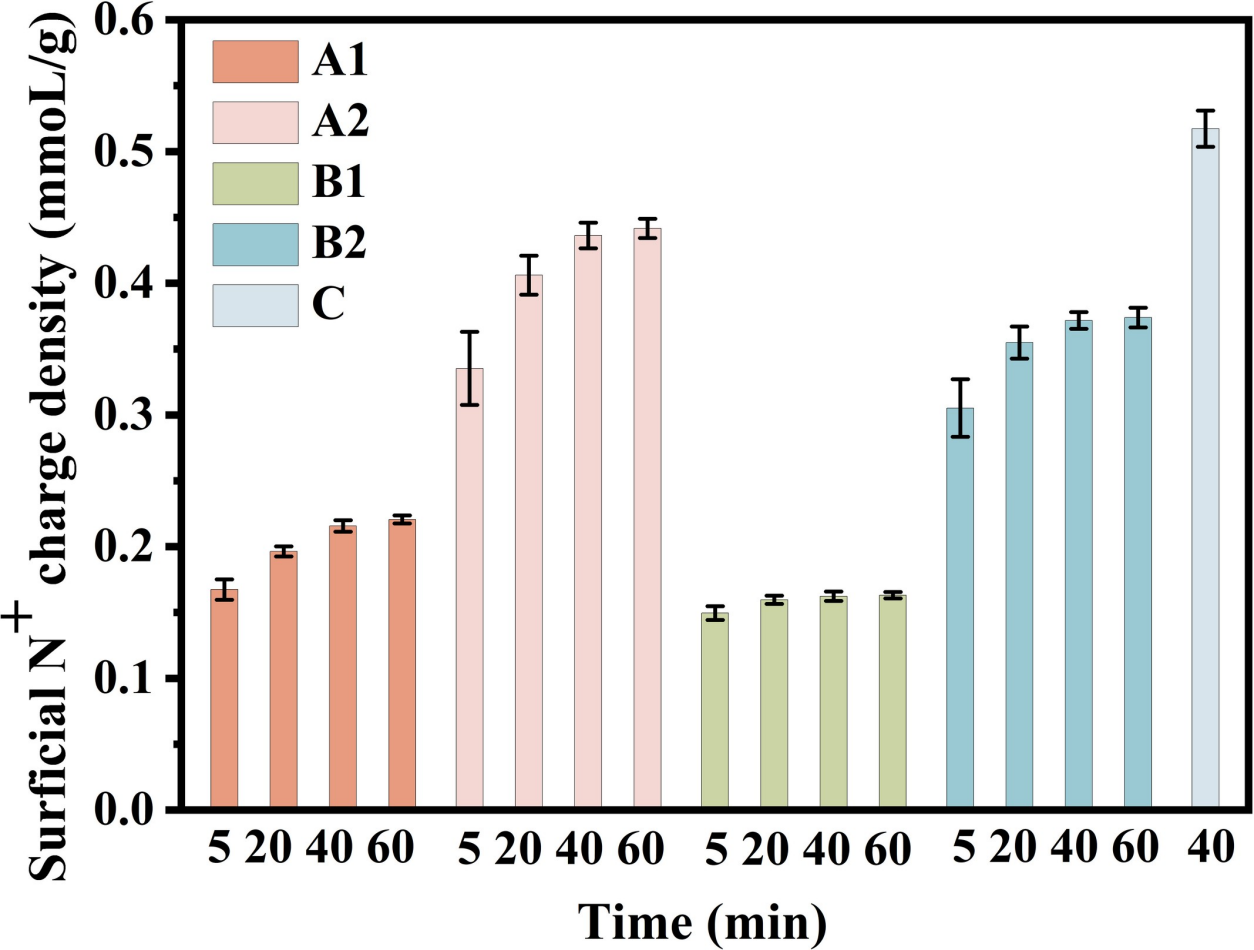
Optimization of detection method of surficial N^+^ charge density.

Fig 1 shows that the alternative detection methods could complete their determinations within 40 min, yet their maximum values vary greatly. In terms of method A1, the detection values increase with reaction time, which shows that the method requires a long detection time for QARs, which is different from the short detection time (instantaneous reaction) for micromolecular QACs [27, 30]. This result hints that it is difficult for the macromolecular TS, mainly containing tetraphenylboron sodium and aluminum hydroxide gel, to permeate into QARs and react with the inner QAs due to the high steric hindrance of TS. Alkaline conditions are essential for the interaction of TS and QAs. Therefore, an earlier alkaline condition in method B (Table 1) is helpful in earlier producing precipitates to block pores and hinder TS further reacting with the inner N^+^ groups, which causes the lower detection values in method B than those in method A. However, the lower detection values in method A1 and B1 than those in method A2 and B2 may be caused by the shaking condition. That is because the latter two could react further with the inner N^+^ groups that were previously hindered by the surficial reaction precipitates, when the precipitates were timely removed through shaking and the resin pores were opened simultaneously. Based on this, B1 is beneficial to more precisely detect the surficial N^+^ groups located on a thinner outer layer of QARs, as we desired. In addition, a higher detection value was found in method C than in methods A and B, which benefits from the lagging alkaline condition and the shaking condition in method C that make the TS easier to permeate QARs and react with the inner QAs. This result reversely proves the previous findings that the earlier alkaline condition and the static condition in method B1 are helpful in minimizing and exactly quantifying the surficial QAs of QARs. In short, TS with high steric hindrance is difficult to permeate into QARs and react with the inner QAs, and most N^+^ active sites are embedded in the interior of QARs and hindered by the instantly formed surficial precipitates, which results in the established method merely detecting the surficial QAs of QARs. Based on the aforementioned conclusions, method B1 is an optimal method to detect the “surficial N^+^ charge density” of QARs, with its earlier alkaline condition and static condition beneficial to exactly and fast complete determination.

A different reaction condition is “step 2” in the methods of A1 and A2 (B1 and B2); an earlier alkaline reaction condition is built in method B than in method A (step 1); method C is a combination of method A2 and method B2.

In addition, the optimum detection time is 40 min for the three resins, including pyridine, styrene or acrylic skeletons (S2 Table in S1 File). Moreover, the best dosage for dry resins is 0.75 g with a relative deviation of < 2.5% (S3 Table in S1 File). More precisely, the best dosage is approximately 0.15 mmol/g of “surficial N^+^ charge density.” We could adjust the sample addition to meet the best detection dosage, for example, adding 10 g sample to achieve the best detection dosage if its real value is only 0.015 mmol/g.

### Verification of surficial N+ charge density

**Fig 2:**
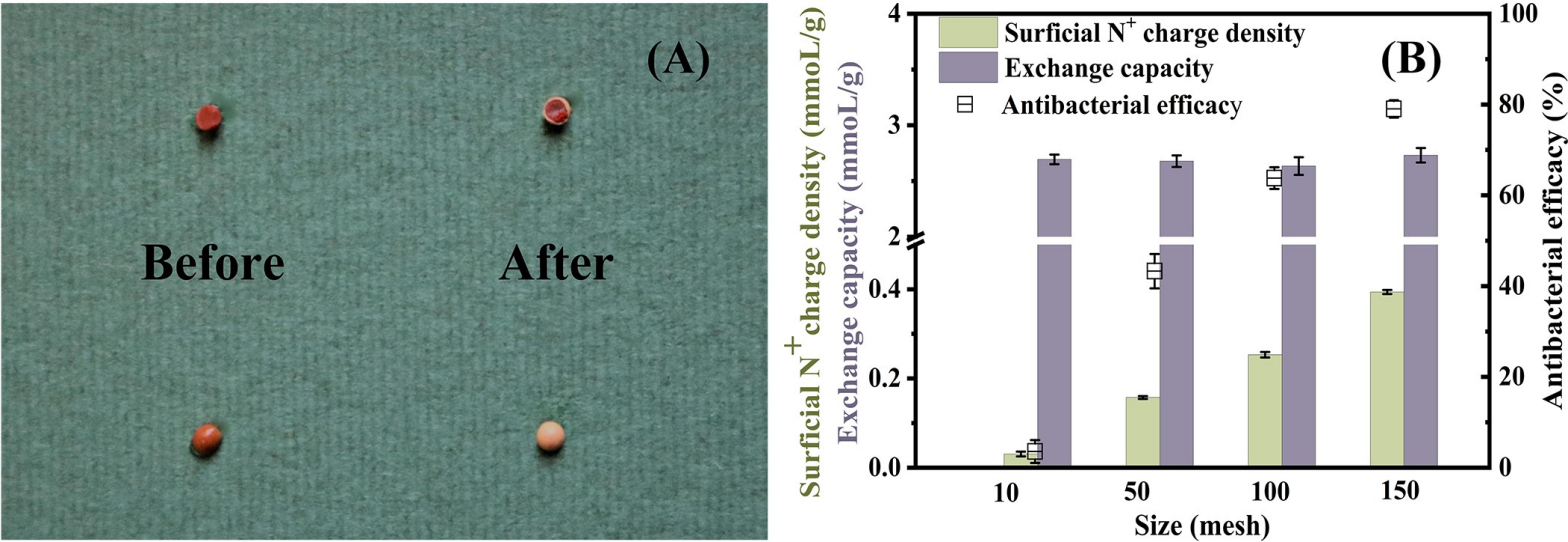
(A) Features of resin D213 before and after detection of surficial N^+^ charge density; (B) changes in surficial N^+^ charge density, exchange capacity and antibacterial efficacy with increasing resin sizes of Py-1C. One resin sphere of D213 was cut into two parts (A), one for showing its interior and the other for showing its surface. The above surficial N^+^ charge densities were detected with the method B1 in Table 1. All antibacterial experiments were performed at least in triplicate, and the error bars indicate the standard deviations from the mean values obtained.

After the detection reaction, the white circle and the white outer surface emerge in Fig 2A, which prove that only the QAs around the thin outer layer of QARs participated in the reaction and produced visible white precipitates. This provides the most direct evidence that the established method merely quantifies the surficial QAs (N^+^ groups). QARs would completely change in color to white with their outer surface and inner pores covered and filled by white reaction precipitates, if TS could react completely with total QAs in QARs. In fact, the TS merely reacts with surficial QAs to form white precipitates, with a white circle emerging around the QAR rim. Furthermore, when the Py-1C resin is milled from 10 to 150 mesh (Fig 2B), we find that its surficial N^+^ charge density and bactericidal ability all increase obviously, but its exchange capacity remains unchanged. The result should be attributed to the smaller particle with a larger specific surface area, causing more inner N^+^ active groups to be exposed to increase the surficial N^+^ charge density and improve the bactericidal ability. Similar results were found in QA-PEI materials with a better antibacterial potency in nanoparticles than in microparticles, and in Polydopamine/Ag_3_PO_4_/Graphene oxide materials with richer reactive oxygen species and higher bacteria-killing effects in smaller particles [24, 31]. The aforementioned results indicate that the surficial N^+^ charge density could be improved sharply when the resin particle was milled into a smaller size with a rising specific surface area and surficial N^+^ groups, which additionally demonstrates that the new method merely detects the exposed surficial QAs.

In addition, the exchange capacities of QARs are higher than their surficial N^+^ charge densities, up to 10–100 times as shown in S1 Table in S1 File, in discordance with their theoretical ratio of 1:1 (one N^+^ group is combined with one Cl^-^, and Cl^-^ indicates the exchange capacities of QARs). This finding demonstrates that the detection method merely involves a small part of QAs in QARs, more likely to be surficial N^+^ groups according to the white reaction circle in Fig 2A. Based on the above conclusions, it is reasonable to define the established method as “surficial N^+^ charge density” for its quantification of only the surficial N^+^ groups, which is different from the exchange capacity of quantifying the total N^+^ groups in QARs and could provide a new parameter for QARs.

### Correlation of surficial N+ charge density and antibacterial performance

**Fig 3:**
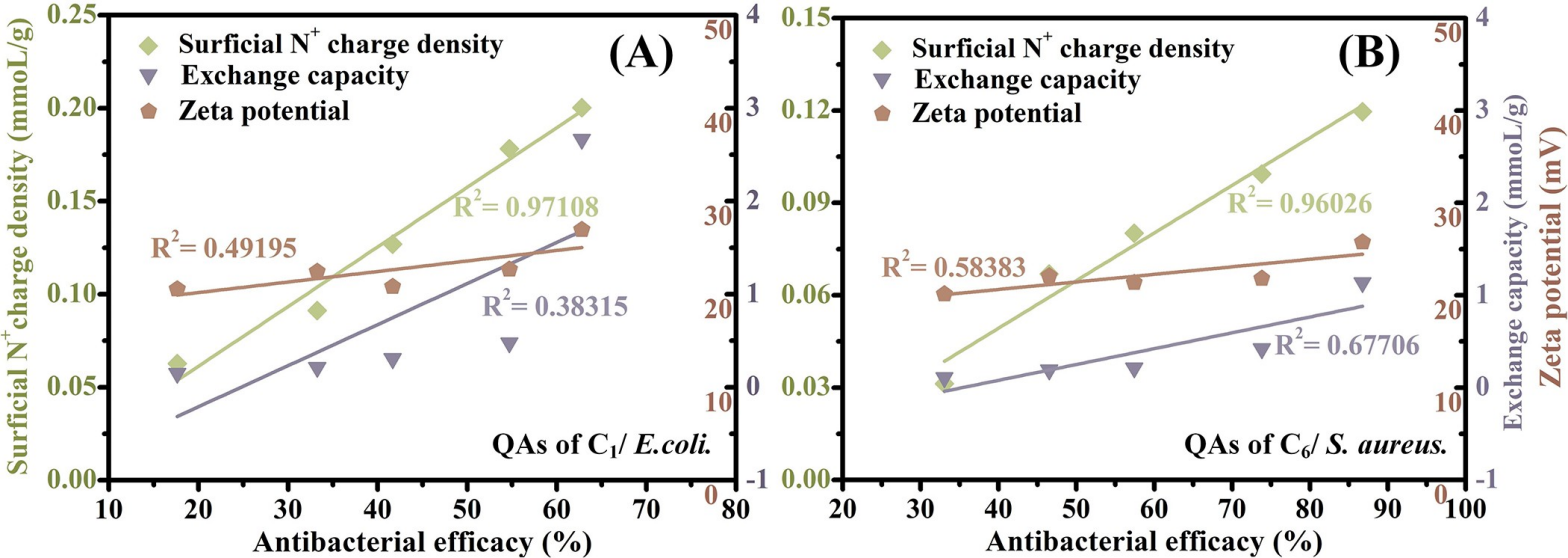
(A) Correlation analysis of antibacterial efficacy with surficial N^+^ charge density, exchange capacity and zeta potential of Py-1C resin; (B) correlation analysis of antibacterial efficacy with surficial N^+^ charge density, exchange capacity and zeta potential of Py-6C resin. QAs: quaternary ammonium groups; “QAs of C_1_/ *E*. *coli*”: QAs with methyl against *E*. *coli*; “QAs of C_6_/ *S*. *aureus*”: QAs with hexyl against *S*. *aureus*. All experiments were performed at least in triplicate.

Fig 3 shows that there is no significant correlation between the antibacterial performance and exchange capacity with the R^2^ values of 0.38 and 0.67, which indicates that partial QAs do not participate in killing bacteria. This result additionally demonstrates that the efficient bactericidal groups are only the surficial QAs, not total QAs. Moreover, the zeta potential has no significant relevance to the antibacterial capacity (R^2^ of 0.49 and 0.58) in Fig 3A and 3B. Jie also found that the antimicrobial resin with a lower zeta potential performed a higher log reduction of *E*. *coli* and *S*. *aureus*, which showed that there was no positive correlation of the bactericidal capacity with the zeta potential [25]. These conclusions should be attributed to the feeble performance of zeta potential in identifying tertiary amine and quaternary ammonium, with two very approximate detection values [24, 32], while the latter has a far higher antibacterial performance than the former. Therefore, the zeta potential is not suitable to exactly indicate the antibacterial capacities of QARs. In addition, when the method of charge density built by Tiller was estimated, we found it rather difficult to fully desorb the fluorescein dye (for the quantification of charge density) from porous and dense-structure resins, failing to exactly quantify and distinguish similar amounts of QAs. However, the surficial N^+^ charge densities all exhibit good correlations with the antibacterial performances in QAs of C_1_ against *E*. *coli* or QAs of C_6_ against *S*. *aureus* (R^2^ of 0.97 and 0.96, respectively, in Fig 3A and 3B). The result demonstrates that the surficial N^+^ charge density is more competent to exactly indicate the bactericidal capacities of QARs, rather than the exchange capacity, zeta potential or charge density. These findings all support that the surficial N^+^ charge density is reliable for estimating the bactericidal properties of QARs with different alkyl-chain QAs against gram-positive or gram-negative bacteria.

Furthermore, the bactericidal property of Py-1C increases with a rising surficial N^+^ charge density when the resin particle was milled into smaller sizes (Fig 2B), which also demonstrates the positive correlation of the antibacterial capacity with surficial N^+^ charge density. The results show that it is possible to improve bactericidal ability by choosing a smaller resin with a higher surficial N^+^ charge density. Similarly, some reported silver nanoparticles or ZnO nanoparticles also exhibited higher antibacterial capacities than those of microparticles [33–35].

**Fig 4:**
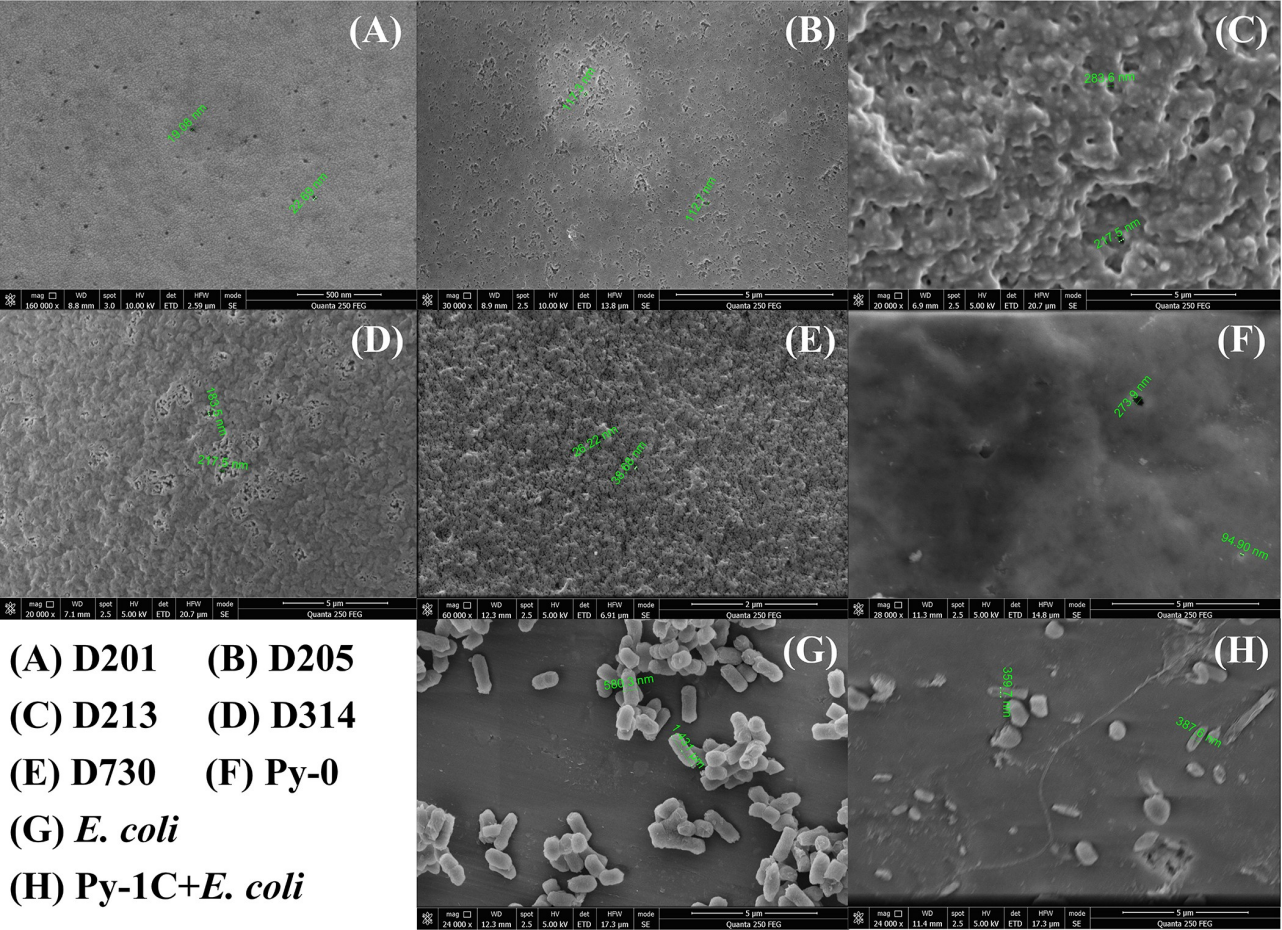
(A) D201 resin; (B) D205 resin; (C) D213 resin; (D) D314 resin; (E) D730 resin; (F) Py-0 resin; (G) *E*. *coli*; (H) conjugates of *E*. *coli* and Py-1C resin. Images were taken using a scanning electron microscope.

Fig 4 shows that it is impossible for larger-size viable germs (diameter of 500‒600 nm, length of 1000‒2000 nm) to permeate into QARs and contact the inner N^+^ groups through smaller-size pores (surficial pores < 300 nm and inner pores < 30 nm in S1 Table in S1 File and Fig 4). This conclusion supports that the efficient disinfection QAs are the surficial N^+^ groups that could directly contact and interact with bacteria. This is consistent with the reported contact-killing bacteria mechanism of QARs (killing bacteria requires a direct interaction of N^+^ groups with bacteria) [21]. In fact, most germs are of micron sizes (diameter of 500‒1000 nm, length of 1000‒2000 nm), such as *E*. *coli*, *P*. *aeruginosa*, *B*. *subtilis*, *S*. *aureus* and *Candida albicans* [14, 16, 36, 37], and most QAR pore sizes are < 200 nm [38–40]. The results show that the efficient bactericidal QAs in QARs are mainly surficial N^+^ groups, which are more correlated to the surficial N^+^ charge density than exchange capacity. The aforementioned conclusions clearly indicate that the most effective strategy for improving antibacterial efficacy is to enrich surficial QAs and improve their bactericidal capacities. These findings disprove the reported traditional synthetic methods of antibacterial resins, for example, prepolymerization or postpolymerization with long-chain alkyl QAs [25, 41], because most painstakingly designed high-cost QAs were embedded in the interior of QARs and were ineffective for killing bacteria. The traditional antibacterial QARs also unavoidably cause a sharp drop in exchange capacity, such as Py-6C (42.6% of Py-1C in S1 Table in S1 File), weakening their potential for removing contaminants, because long-chain alkyls of higher steric hindrance are harmful to grafting QAs. It is wise to prepare an antibacterial resin with two-step quaternization, one for surficial grafting with long-chain alkyls (for better bactericidal performance) and the other for total grafting with short-chain alkyls (for richer QAs). New resin with this design has been put into practice, which exhibits better antibacterial capacity, with a higher surficial N^+^ charge density (40%) and exchange capacity (60%) than that of traditional resin Py-6C in our further study.

### Applicability of surficial N+ charge density

**Fig 5:**
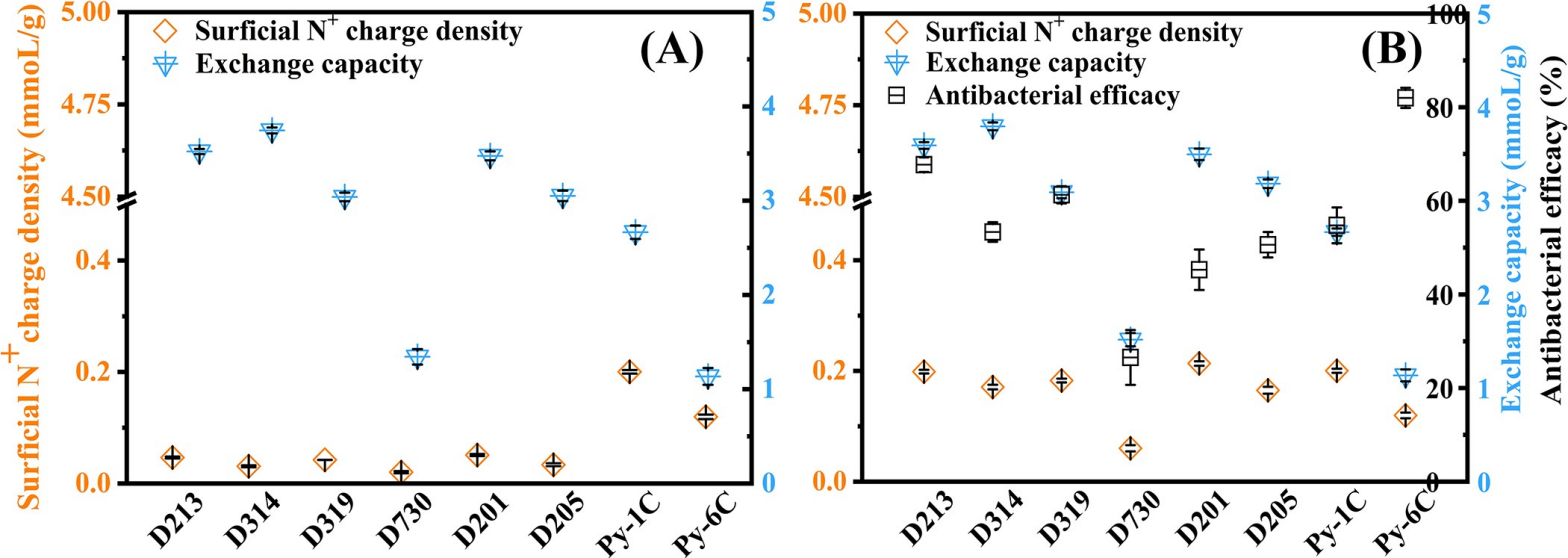
(A) Comparison of values of surficial N^+^ charge densities and exchange capacities in different quaternary ammonium resins (QARs); (B) estimation application of surficial N^+^ charge density indicating antibacterial efficacy in commercial QARs. Particle sizes of QARs are shown in S1 Table in S1 File (A); Particle sizes of QARs are of 80‒100 mesh (B). All antibacterial experiments were performed at least in triplicate, and the error bars indicate the standard deviations from the mean values obtained.

We chose eight resins to test the applicability of “surficial N^+^ charge density” in commercial QARs, including acrylic, styrene and pyridine resin skeletons, with different QAs, cross-linkages of 10%‒35%, particle size ranges of 10‒100 mesh, and average pore sizes of 10‒30 nm (S1 Table in S1 File). Fig 5A shows that the surficial N^+^ charge densities of QARs are far below their exchange capacities, while their theoretical ratio should be 1:1 as described before. The result shows that only partial QAs (exposed surficial QAs) could be detected, which demonstrates that the established method is efficient and competent fully for these common commercial QARs. The new method was suitable for the above resins with surficial pore sizes of < 300 nm (Fig 4), which hints that other similar quaternary ammonium materials with similar pore sizes might adopt it, if an obvious gap value is found between the surficial N^+^ charge density and exchange capacity. From Fig 5B, the antibacterial efficacies of acrylic resins D213, D314, D319 and D730 are shown to keep pace with their surficial N^+^ charge densities but not with their exchange capacities, which indicates that the new method works well in practical applications. Inconsistently, the D213, D201 and Py-1C resins have a similar surficial N^+^ charge density but exhibit a disorder in antibacterial performance, which should be interfered by their different skeletons. However, an inverse result was found in Py-1C and Py-6C (D201 and D205), which probably be caused by their different bactericidal QAs (QAs with C_6_ in the latter exhibited higher antibacterial superiority than QAs with C_1_ in the former). Likewise, the reported optimal bactericidal alkyls of QARs were from C_6_ to C_12_, with better antibacterial performance than C_1_ [21, 42, 43]. In short, we have demonstrated that it is acceptable for most commercial resins with a wide range of parameters to detect their surficial N^+^ charge densities through the established method. However, surficial N^+^ charge density is more suitable to estimate the potential antibacterial capacities for the QARs with similar skeletons and similar QAs, yet incompetent for the QARs with diverse skeletons or different alkyl-chain QAs.

## Conclusions

In summary, the “surficial N^+^ charge density” of QARs is first proposed and demonstrated to efficiently indicate the antibacterial capacity, and its detection method is a simple in-situ determination, better than the reported charge density or zeta potential. The detection mechanism of surficial N^+^ charge density depends on the colloidal solution of TS, which merely reacts with the exposed surficial QAs of QARs because of its high steric hindrance and its reaction precipitates. Meanwhile, the results showed that the antibacterial capacity of QARs has no significant relevance to their exchange capacity or zeta potential. The established method was also verified to meet most commercial resins to detect their surficial N^+^ charge densities, thus estimating their antibacterial efficacies against both gram-negative and gram-positive bacteria in practical applications. Furthermore, the surficial N^+^ charge density was proved competent for selecting a highly efficient antibacterial resin from the QARs with similar skeletons and similar QAs.

This study provides a clear direction for improving the antibacterial capacity of QARs by enriching the surficial N^+^ charge density, selecting higher efficient bactericidal QAs and decreasing resin particle size, which could overcome the limited disinfection property and low exchange capacity of traditional antibacterial resins. All the progress we make will boost the disinfection development of QARs in water environments. High-efficiency antibacterial QARs might be a promising disinfection alternative in water treatments, especially as a disinfection unit of drinking water purifiers, due to no DBPs and a lower cost for their reusability.

## Supporting information

S1 File (PDF)

S1 Text (TXT)

S1 Graphical abstract (TIF)
