# Supplementary material for: Surficial N+ charge density indicating antibacterial capacity of quaternary ammonium resins in water environment
Source: PLoS One. 2020 Sep 30;15(9):e0239941. doi: 10.1371/journal.pone.0239941 (PMC7526928; doi:10.1371/journal.pone.0239941)

Supporting Information for

“Surficial N^+ charge density indicating antibacterial capacity of quaternary ammonium resins in water environment”

Huaicheng Zhang, Aimin Li*, Kaiqin Bian, Shanqi Shen, Peng Shi

Affiliations of authors:

[a]. State Key Laboratory of Pollution Control and Resource Reuse, School of the Environment, Nanjing University, Nanjing 210023, P.R. China.

[*]**Corresponding author**

Addresses:

State Key Laboratory of Pollution Control and Resource Reuse, School of the Environment, Nanjing University, 163 Xianlin Avenue, Nanjing 210023, China.

Phone: +86-25-89680507

Fax: +86-25-896800507

Email: liaimingroup@nju.edu.cn

Submitted to *PLOS ONE*

CONTENTS

1. Tables

S1 Table. Characteristic parameters of QARs.

S2 Table. Optimum detection time of surficial N^+ charge density.

S3 Table. Optimum detection dosage of surficial N^+ charge density.

2. Figures

S1 Fig. FT-IR spectra of resins of Py-0, Py-1C and Py-6C.

Tables

S1 Table. Characteristic parameters of QARs

Resins	Skeleton	Alkyl chain length	Cross-linkage	Size (um)	Average pore diameter (nm)/BET	Strong-base group exchange capacity (mmol/g)	Surficial N^+ charge density (mmol/g)
Py-1C	pyridine	1	25%	80~100 Mesh / 176um	16.09	2.6657	0.1897±1.16%
Py-6C	pyridine	6	25%	80~100 Mesh / 176um	15.62	1.1359	0.1196±3.39%
D201	styrene	1	20%	30~50 Mesh / 469um	13.29	3.4752	0.05111±3.69%
D205	styrene	1	20%	30~50 Mesh / 448um	13.95	3.0513	0.03372±4.51%
D213	acrylic	1	20%	30~50 Mesh / 479um	16.76	3.5217	0.04671±3.68%
D314	acrylic	1	10%	10~30 Mesh / 933um	29.17	3.7452	0.03091±3.82%
D319	acrylic	1	15%	30~50 Mesh / 473um	21.48	3.0396	0.04232±3.43%
D730	acrylic	1	35%	50~80 Mesh / 316um	10.93	1.3439	0.02034±4.67%

S2 Table. Optimum detection time of surficial N^+ charge density

resins	5min	20min	40min	60min	90min
D201	0.1637	0.1908	0.1993	0.1986	0.1998
D213	0.1608	0.1727	0.1763	0.1773	0.1771
Py-1C	0.1583	0.1804	0.1872	0.1879	0.1873

Particle sizes of resins of D201, D213 and Py-1C are of 80~100 mesh.

S3 Table. Optimum detection dosage of surficial N^+ charge density

resins	0.25g	0.5g	0.75g	1.00g	1.50g
D201	0.05103±16.25%	0.1072±4.78%	0.1547±2.18%	0.2084±1.56%	0.3037±0.49%
D213	0.04678±17.68%	0.09159±5.81%	0.1416±2.05%	0.1859±1.29%	0.2789±0.64%
Py-1C	0.04709±14.46%	0.09337±5.39%	0.1429±1.89%	0.1897±1.16%	0.2876±0.72%

Particle sizes of resins of D201, D213 and Py-1C are of 80~100 mesh.

S1 Fig. FT-IR spectra of resins of Py-0, Py-1C and Py-6C

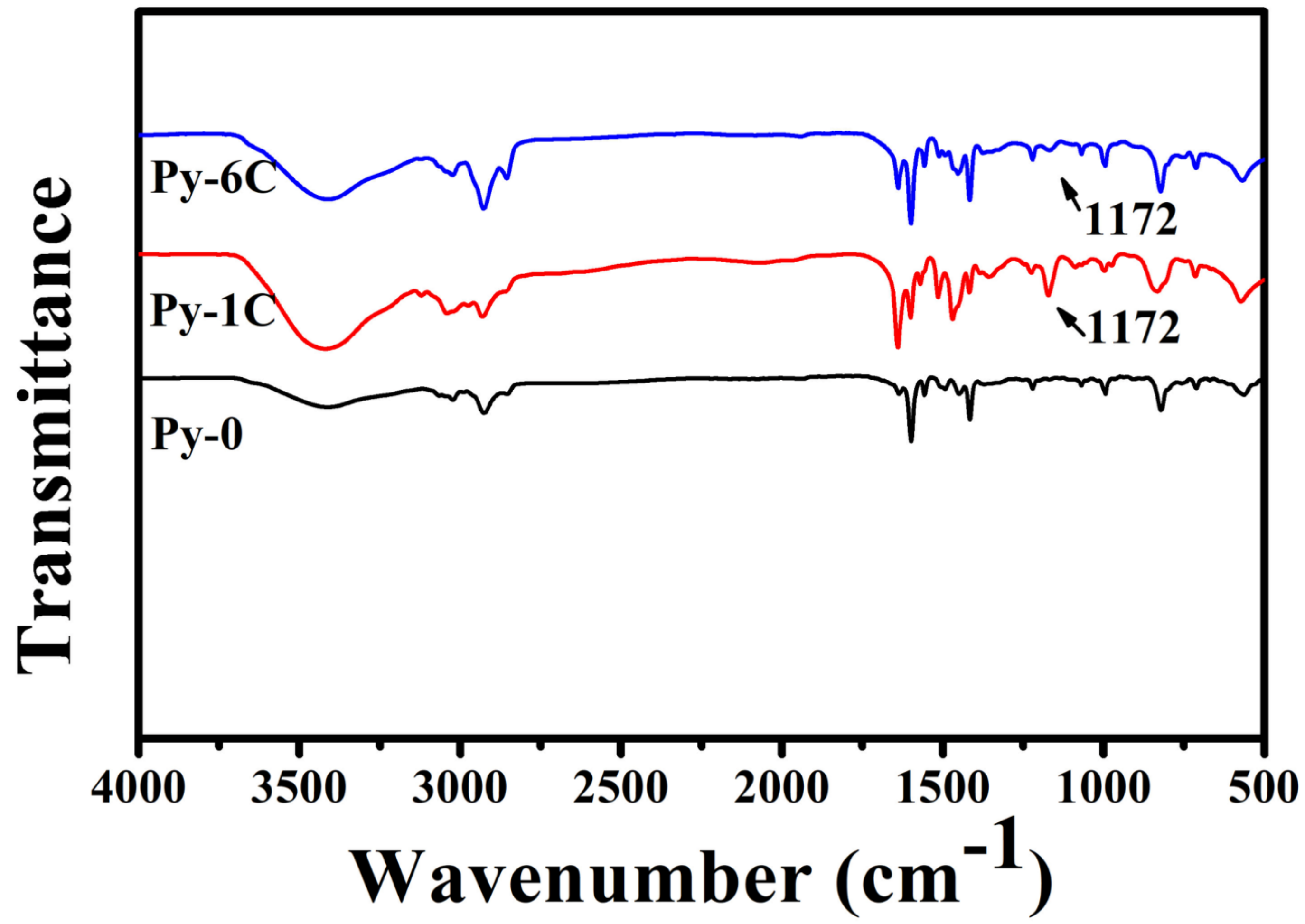

Supplement: S1 File — (PDF) [file pone.0239941.s001.pdf]
